# Inhibition of p70 S6 kinase activity by A77 1726 induces autophagy and enhances the degradation of superoxide dismutase 1 (SOD1) protein aggregates

**DOI:** 10.1038/s41419-018-0441-0

**Published:** 2018-03-14

**Authors:** Jing Sun, Yarong Mu, Yuanyuan Jiang, Ruilong Song, Jianxin Yi, Jingsong Zhou, Jun Sun, Xinan Jiao, Richard A. Prinz, Yi Li, Xiulong Xu

**Affiliations:** 1 Institute of Comparative Medicine, Yangzhou University, Yangzhou, 225009 Jiangsu Province China; 2 College of Veterinary Medicine, Yangzhou University, Yangzhou, 225009 Jiangsu Province China; 3 Department of Physiology, Kansas City University of Medicine and Biosciences, Kansas City, MO 64106 USA; 4 Department of Medicine, University of Illinois at Chicago, Chicago, IL 60612 USA; 5 Jiangsu Co-innovation Center for Prevention and Control of Important Animal Infectious Diseases and Zoonosis, Yangzhou University, Yangzhou, 225009 Jiangsu Province China; 6 Department of Surgery, NorthShore University Health System, Evanston, IL 60201 USA; 7 Lester and Sue Smith Breast Center, Baylor College of Medicine, Houston, TX 77030 USA; 8 Department of Cell and Molecular Medicine Rush University Medical Center, Chicago, IL 60612 USA

## Abstract

Autophagy plays a central role in degrading misfolded proteins such as mutated superoxide dismutase 1 (SOD1), which forms aggregates in motor neurons and is involved in the pathogenesis of amyotrophic lateral sclerosis (ALS). Autophagy is activated when UNC-51-like kinase 1 (ULK1) is phosphorylated at S555 and activated by AMP-activated protein kinase (AMPK). Autophagy is suppressed when ULK1 is phosphorylated at S757 by the mechanistic target of rapamycin (mTOR). Whether p70 S6 kinase 1 (S6K1), a serine/threonine kinase downstream of mTOR, can also regulate autophagy remains uncertain. Here we report that inhibition of S6K1 by A77 1726, the active metabolite of an anti-inflammatory drug leflunomide, induced mTOR feedback activation and ULK1^S757^ phosphorylation in NSC34 cells, a hybrid mouse motoneuron cell line. Unexpectedly, A77 1726 did not suppress but rather induced autophagy by increasing AMPK^T172^ and ULK1^S555^ phosphorylation. Similar observations were made with PF-4708671, a specific S6K1 inhibitor, or with S6K1 siRNA. Further studies showed that A77 1726 induced AMPK phosphorylation by activating the TGF-β-activated kinase 1 (TAK1). Functional studies revealed that A77 1726 induced co-localization of mutant SOD1^G93A^ protein aggregates with autophagosomes and accelerated SOD1^G93A^ protein degradation, which was blocked by inhibition of autophagy through autophagy-related protein 7 (ATG7) siRNA. Our study suggests that S6K1 inhibition induces autophagy through TAK1-mediated AMPK activation in NSC34 cells, and that blocking S6K1 activity by a small molecule inhibitor such as leflunomide may offer a new strategy for ALS treatment.

## Introduction

Amyotrophic lateral sclerosis (ALS) is the most common form of adult-onset motoneuron degenerative disease characterized by the selective loss of motoneurons in the ventral horn of the spinal cord, the cerebral cortex, and brainstem nuclei^1, 2^. Approximately 90% of ALS is sporadic and does not have an apparent genetic linkage. The remaining 10% is familial and these patients carry a mutant gene^3^. Superoxide dismutase 1 (*SOD1*) was the first mutated gene to be discovered in familial ALS about two decades ago^4–6^. Mutant SOD1 proteins are prone to misfolding and forming aggregates in motoneurons. Several other genes, including TAR DNA-binding protein 43 (*TDP-43*), Fused in Sarcoma/Translocated in Sarcoma (*FUS/TLS*), and chromosome 9 open reading frame 72 (*C9ORF72*), have also been found to be mutated in familial ALS patients^3^. The products of these genes, TDP-43, FUS, and DPR (dipeptide repeat), can also form aggregates that cannot be easily degraded. The presence of protein aggregates in the cytosol activates macroautophagy (often referred as autophagy), a cellular process involved in degrading long-lived proteins and damaged organelles such as mitochondria^3, 7, 8^. Inability to remove protein aggregates leads to cell death and neurodegeneration^3, 7, 8^. Recent studies have shown that several genes involved in autophagy, including p62/SQSTM1 (SQSTM1), ubiquilin 2 (UBQLN2), optineurin 1 (OPTN1), TANK-binding kinase 1 (TBK1), are mutated in familial ALS patients^8–10^. Therapeutic intervention to activate autophagy and subsequently decrease the load of protein aggregates and oligomers has alleviated ALS in preclinical studies^8–10^. Better understanding of the regulation of autophagy will help designing novel therapeutic strategies to treat this fatal disease.

**Fig. 1:**
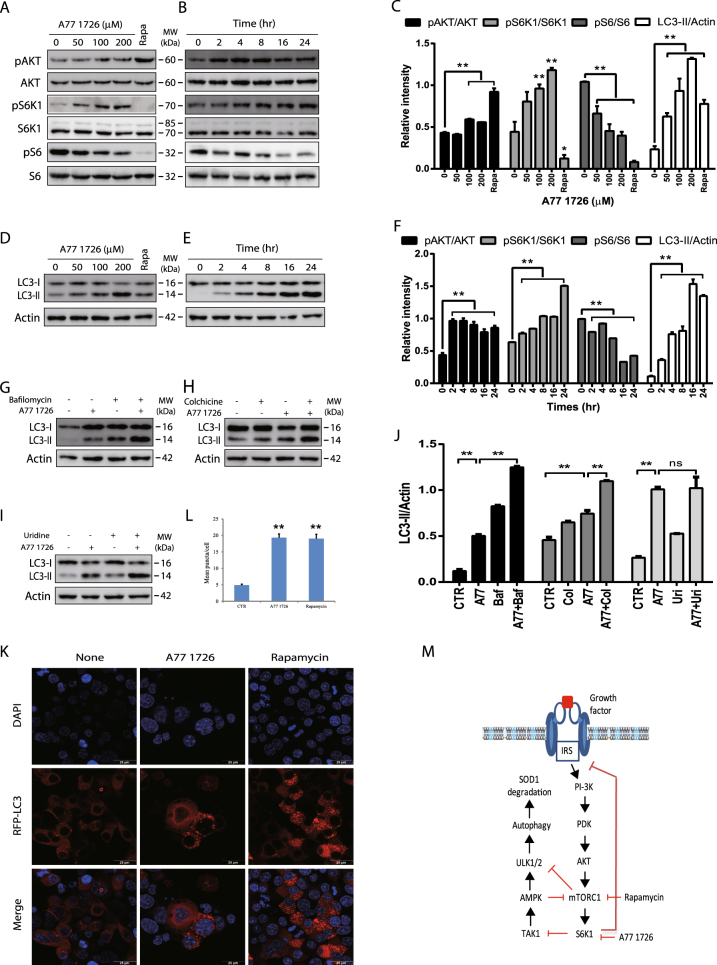
The effect of A77 1726 on the feedback activation of the PI-3 kinase pathway and autophagy. **a**–**f** The effect of A77 1726 on the feedback activation of the PI-3 kinase pathway and LC3-II lipidation. NSC34 cells were incubated in complete DMEM medium in the absence or presence of the indicated concentrations of A77 1726 for 16 h (**a**, **c**, **d**) or were incubated in the presence of A77 1726 (200 μM) for the indicated time (**b**, **e**, **f**). Rapamycin (50 nM) was included as a positive control (**a**, **d**, **c**). Cell lysates were analyzed for the feedback activation of the PI-3 kinase pathway (**a**, **b**) or for LC3-II lipidation (**c**, **d**) by western blot with the indicated antibodies. **g**, **h** The effect of bafilomycin and colchicine on LC3-II lipidation. NSC34 cells were incubated in complete DMEM medium in the absence or presence of A77 1726 (200 μM) minus or plus bafilomycin (100 nM) (**g**, **j**) or colchicine (5 μM) (**h**, **j**) for 16 h. Cell lysates were analyzed for LC3 and actin expression by western blot. **i**, **j** Inability of uridine to block A77 1726-induced LC3-II lipidation. NSC34 cells were incubated in complete DMEM medium in the absence or presence of A77 1726 (200 μM) minus or plus uridine (200 μM) for 16 h. Cell lysates were analyzed for LC3-II lipidation and actin expression by western blot. The expression levels were analyzed by quantification of the density of the protein bands with NIH Image-J software and presented as bar graphs (**c**, **f**, **j**). LC3 lipidation was analyzed by comparing the density of LC3-II with β-actin. The data in Fig. 1c, f, j and the remaining Image-J-derived data in all other figures are the mean ± SD from three experiments**. k**, **l** NSC34 cells were transfected with the expression vector pmLC3-RFP. The cells were left untreated or treated with A77 1726 (200 μM) or rapamycin (50 nM) for 16 h. Autophagosomes were visualized under a confocal microscope (**k**). The puncta of autophagosomes were counted under a fluorescent microscope and plotted in a bar graph with statistical analysis (**l**). *p < 0.05; **p < 0.01. **m** Schematic model of A77 1726-induced autophagy. Inhibition of S6K1 activity leads to TAK1 activation, which activates AMPK. AMPK phosphorylates ULK1^S555^ and activates it. Inhibition of S6K1 by A77 1726 leads to the feedback activation of the PI-3 kinase pathway, as evidenced by increased AKT and S6K1 phosphorylation. Rapamycin also induces the feedback activation of the PI-3 kinase pathway. However, rapamycin targets mTOR, leading to decreased S6K1 phosphorylation

Autophagy is initiated by the class III PI-3 kinase (Vps34) that complexes with Beclin-1 and ATG14 to trigger the nucleation of the membrane from the endoplasmic reticulum^11^. On the other hand, autophagy is inhibited by activation of the class I PI-3 kinase pathway through mTOR, a serine/threonine kinase that phosphorylates ULK1/2 and inhibits the assembly of the autophagic machinery (Fig. 1m)^11^. AMPK activation due to energy stress leads to ULK1^S555^ phosphorylation and activation, thus directly initiating autophagy^12–14^. mTOR inhibitors and AMPK activators have been sought as autophagy inducers to degrade protein aggregates in motor neurons and to ameliorate ALS progression^8^. S6K1 is a serine/threonine kinase phosphorylated and activated by mTOR, and is overexpressed and highly activated in the spinal cord of ALS patients and in transgenic mice with the SOD1^G93A^ gene^15, 16^. Whether S6K1 inhibition can induce autophagy and accelerate mutant SOD1 protein degradation has not been studied.

TAK1 is a serine/threonine kinase activated by IL-1 and TGF-β receptors, Toll-like receptors, CD40, and B cell receptor^17–19^. TAK1 plays important roles in cell survival, differentiation, apoptosis, and inflammatory responses. Recent studies have shown that TAK1 inactivation mutations cause frontometaphyseal dysplasia^20^ and cardiospondylocarpofacial syndrome^21^. TAK1 phosphorylates and activates several intracellular kinases, including p38, c-Jun N-terminal kinase (JNK), and I-kappa B kinase complex (IKK)^22–25^. In addition, TAK1 also activates the tumor suppressor protein LKB1, leading to AMPK^T172^ phosphorylation and activation (Fig. 1m)^26^. Inokuchi-Shimizu et al^27^. reported that TAK1 is required for starvation-induced AMPK and ULK1 phosphorylation and activation, and plays a critical role in inducing autophagy. Moreover, TAK1 deficiency partially blocks rapamycin-induced autophagy in hepatocytes^27^. Mechanisms by which TAK1 promotes autophagy and its involvement in clearing protein aggregates remain to be defined.

Leflunomide (Arava^TM^) is an anti-inflammatory drug approved for treating rheumatoid arthritis (RA). A77 1726 and its parental drug, leflunomide, inhibit tyrosine phosphorylation and pyrimidine nucleotide synthesis^28–35^. The ability of A77 1726 to inhibit the activity of dihydroorotate dehydrogenase (DHO-DHase), a rate-limiting enzyme in pyrimidine nucleotide synthesis, is much stronger than its ability to inhibit the activity of protein tyrosine kinases such as p56^lck^, p59^fyn^, and platelet-derived growth factor (PDGF) receptor^28–32^. Our recent study showed that leflunomide and its active metabolite A77 1726 directly inhibit the activity of purified p70 S6 kinase (S6K1) in an in vitro kinase assay. Inhibition of S6K1 in an A375 melanoma cell line by A77 1726 leads to the feedback activation of the PI-3 kinase pathway as evidenced by increased AKT and S6K1 phosphorylation but modestly or weakly decreased S6 phosphorylation (Fig. 1m)^36^. Here we report that A77 1726 induced autophagy and SOD1 degradation in NSC34 cells through TAK1-induced AMPK activation (Fig. 1m).

## Results

### Autophagy induction by A177 1726 in NSC34 cells

**Fig. 2:**
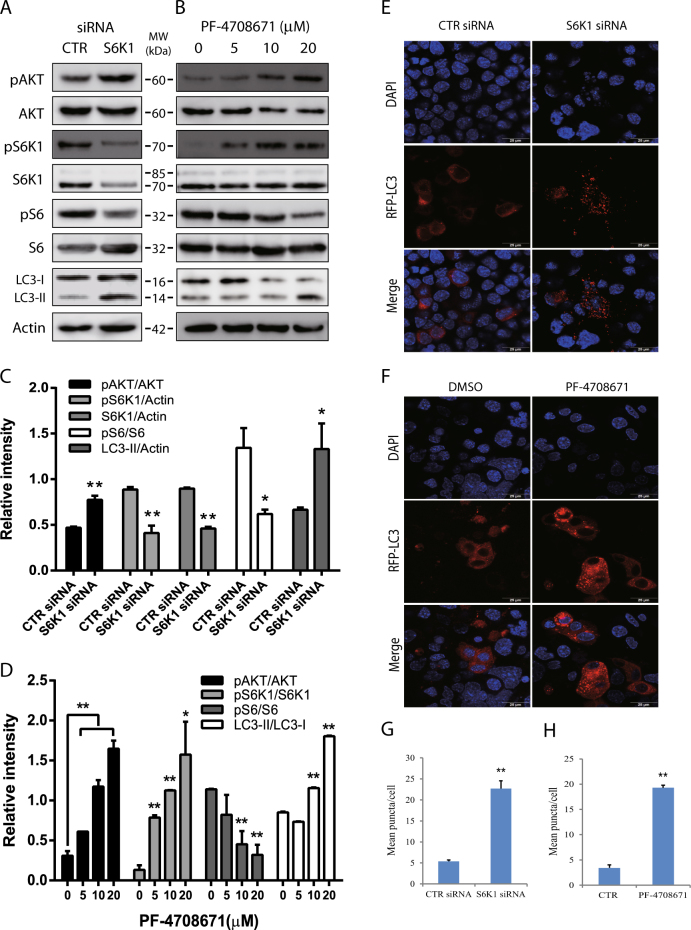
S6K1 inhibition induces autophagy. **a**, **c** The effect of S6K1 knockdown on LC3-II lipidation. NSC34 cells were transfected with scrambled or S6K1 siRNA (2.5 nmole each). After incubation for 48 h, cell lysates were prepared and analyzed for total and phosphorylated proteins by western blot. **b**, **d** The effect of the S6K1 inhibitor on LC3-II expression. NSC34 cells seeded in 6-well plates were incubated in complete DMEM medium in the absence or presence of the indicated concentrations of PF-4708671 for 16 h. Cell lysates were analyzed for total and phosphorylated proteins by western blot. The expression levels were analyzed by quantification of the density of the protein bands with NIH Image-J software and presented as bar graphs (**c**, **d**). **e** The effect of S6K1 knockdown on autophagosome formation. NSC34 cells seeded on the coverslips were first transfected with scrambled or S6K1 siRNA (2.5 nmole each). After incubation overnight, the cells were transfected with pmLC3-RFP expression vector. After incubation for 48 h, the cells were fixed in methanol and visualized for autophagosomes under a confocal fluorescent microscope. **f** The effect of the S6K1 inhibitor on LC3-II expression. NSC34 cells seeded on coverslips were transfected with LC3-RFP expression vector. After incubation for 24 h, the cells were treated with DMSO (0.2%) or PF-4708671 (20 μM) for 16 h. Cells were fixed and analyzed for autophagosomes under a fluorescent microscope. **g**, **h** The puncta of autophagosomes were counted under a fluorescent microscope and plotted in a bar graph with statistical analysis. *p < 0.05; **p < 0.01

Consistent with our prior observations^36^, A77 1726 increased AKT^S473^ and S6K1^T389^ phosphorylation in a dose-dependent manner in NSC34 cells (Fig. 1a,c). A77 1726 rapidly induced AKT^S473^ and S6K1^T389^ phosphorylation, as soon as 2 h after exposure to A77 1726 (Fig. 1b,f). A77 1726 modestly inhibited S6 phosphorylation due to S6K1 hyperactivation (Fig. 1a, b), a phenomenon consistent with the observation made with other S6K1 inhibitors such as PF-4708671 as shown below (Fig. 2b). We next tested if mTOR feedback activation by A77 1726 led to the inhibition of autophagy. Surprisingly, A77 1726 increased LC3-II lipidation in a dose- (Fig. 1d) and time-dependent (Fig. 1e) manner in NSC34 cells. Rapamycin included as a positive control modestly increased LC3-II levels (Fig. 1d). Increased LC3-II lipidation was not due to the stall of autophagy flux since combination of A77 1726 with bafilomycin (Fig. 1g,j) or colchicine (Fig. 1h,j) increased the levels of LC3-II and increased the ratios of LC3-II to LC-I, compared to bafilomycin or colchicine alone. A77 1726 inhibits pyrimidine nucleotide synthesis by inhibiting DHO-DHase activity^28, 29^. Uridine can be used to normalize pyrimidine nucleotide levels in vitro and in vivo^28, 29^. We found that exogenous uridine (200 μM) was unable to block A77 1726-induced increase of LC3-II levels (Fig. 1i, j), suggesting that increased LC3 lipidation by A77 1726 was not due to its inhibitory effect on pyrimidine nucleotide synthesis. Confocal microscopic fluorescence analysis revealed that LC3 formed autophagosomes in NSC34 cells in the presence of A77 1726 or rapamycin (Fig. 1k). Statistical analysis revealed that the number of autophagosome puncta was significantly higher in NSC34 cells treated with A77 1726 or rapamycin than that in the untreated controls (Fig. 1l).

### Autophagy induction by suppression of S6K1 expression or activity

We next tested if S6K1 siRNA also induced autophagy in NSC34 cells. As shown in Fig. 2a, c, S6K1 siRNA reduced S6K1 expression and S6 phosphorylation but increased AKT phosphorylation and LC3-II lipidation. PF-4708671, a specific inhibitor of S6K1, modestly inhibited S6 phosphorylation but induced the feedback activation of the PI-3 kinase pathway, evidenced by increased AKT and S6K1 phosphorylation (Fig. 2b, d). Consistently, PF-4708671 increased the ratio of LC3-II/LC3-I in a dose-dependent manner in NSC34 cells (Fig. 2b, d). Both S6K1 knockdown and PF-4708671 increased the number of LC3-RFP puncta (Fig. 2e, f) in NSC34 cells. The number of autophagosome puncta was significantly higher in NSC34 cells with S6K1 knockdown or treated with PF-4708671 than their corresponding controls (Fig. 2g, h).

### Inhibition of S6K1 activity leads to AMPK and ULK1 phosphorylation

**Fig. 3:**
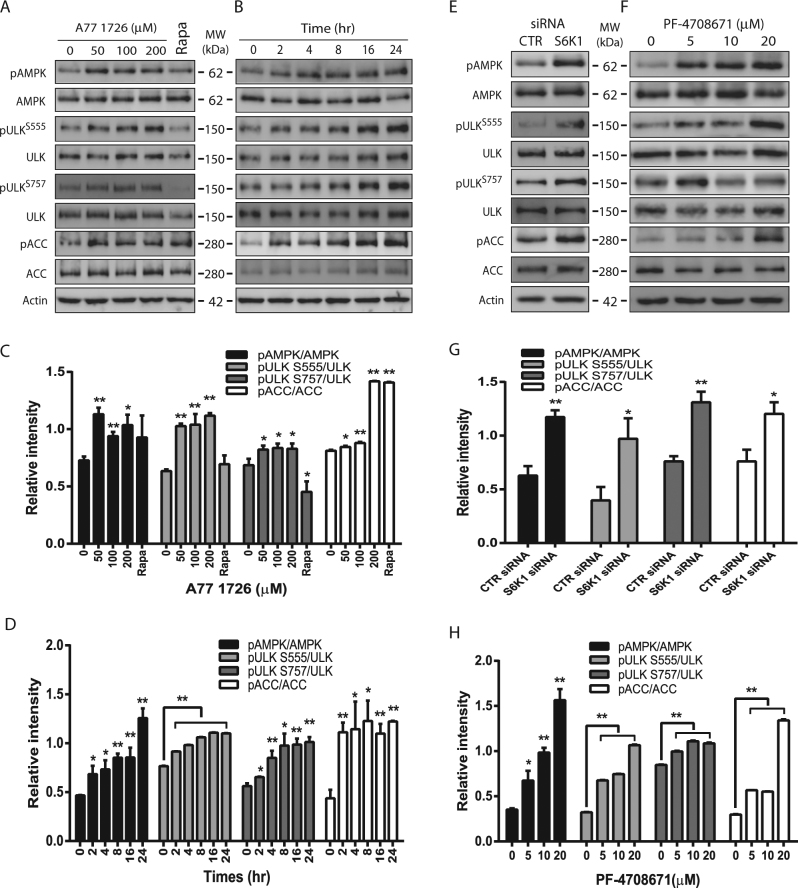
AMPK and ULK1 phosphorylation by S6K1 inhibition. NSC34 cells were incubated in complete DMEM medium in the absence or presence of the indicated concentrations of A77 1726 for 16 h (**a**, **c**) or in the presence of A77 1726 (200 μM) for the indicated time (**b**, **d**). NSC34 cells were transfected with S6K1 siRNA and incubated for 48 h (**e**, **g**) or were treated with the indicated concentrations of PF-4708671 for 16 h (**f**, **h**). Cell lysates were analyzed for total and phosphorylated proteins by western blot. The expression levels were analyzed by quantification of the density of the protein bands with NIH Image-J software and presented as bar graphs (**c**, **d**, **g**, **h**). *p < 0.05; **p < 0.01, compared to the untreated control

AMPK phosphorylates ULK1^S555^ and induces autophagy^12, 37^. Here we tested if A77 1726 induced autophagy by phosphorylating and activating AMPK and ULK1. A77 1726 significantly increased AMPK^T172^, ULK1^S555^, and acetyl-CoA carboxylase (ACC^S79^) (another substrate of AMPK) phosphorylation in NSC34 cells even at 50 μM (Fig. 3a, c) and in a time-dependent (Fig. 3b, d) manner. mTOR is activated by A77 1726 due to the feedback activation of the PI-3 kinase pathway^36^. A77 1726 induced ULK1^S757^ phosphorylation in a dose- and time-dependent manner (Fig. 3a–d). Rapamycin did not increase AMPK^T172^ and ULK1^S555^ phosphorylation but suppressed ULK1^S757^ phosphorylation (Fig. 3a,c). Consistent with these observations, suppression of S6K1 expression by S6K1 siRNA (Fig. 3e, g) or inhibition of S6K1 activity by PF-4708671 (Fig. 3f, h) led to increased AMPK^T172^, ULK1^S555^, ULK1^S757^, and ACC^S79^ phosphorylation.

### Evidence that AMPK mediates A77 1726-induced ULK1 phosphorylation and autophagy

**Fig. 4:**
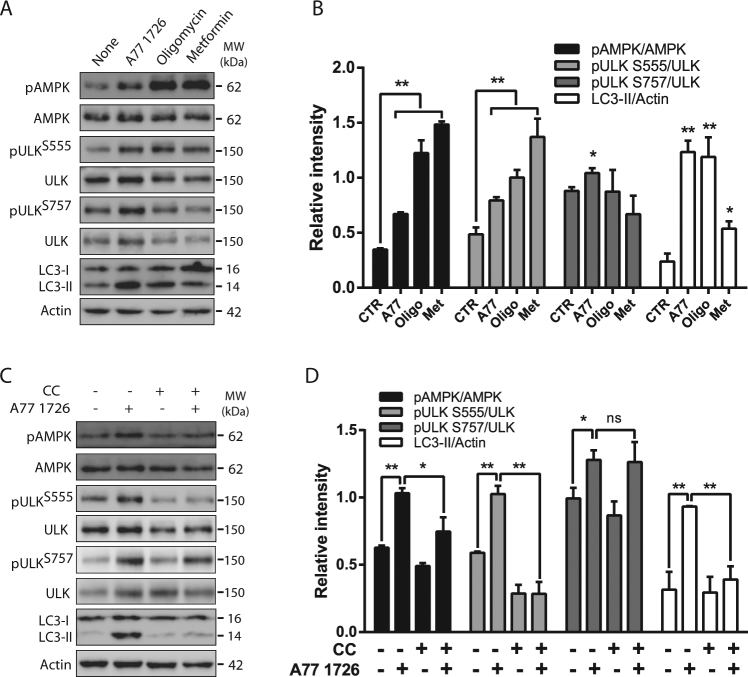
Role of AMPK in A77 1726-induced autophagy. NSC34 cells were incubated in the absence or presence of A77 1726 (200 μM), oligomycin (5 μM) or metformin (10 mM) for 16 h (**a**, **b**) or were incubated in the absence or presence of A77 1726 (200 μM) and/or compound C (5 μM) for 16 h (**c**, **d**). Cell lysates were prepared and analyzed for total and phosphorylated proteins by western blot. The expression levels were analyzed by quantification of the density of the protein bands with NIH Image-J software and presented as bar graphs (**b**, **d**). *p < 0.05; **p < 0.01

We then determined if AMPK activation by A77 1726 was indeed responsible for ULK1 phosphorylation and autophagy. As shown in Fig. 4a, b, two AMPK activators, oligomycin and metformin, induced AMPK^T172^ and ULK1^S555^ phosphorylation but had no effect on ULK1^S757^ phosphorylation. Oligomycin was more potent than metformin in inducing LC3-II lipidation. Compound C (CC), an inhibitor of AMPK, did not significantly inhibit A77 1726-induced ULK1^S757^ phosphorylation but blocked A77 1726-induced AMPK^T172^ and ULK1^S555^ phosphorylation as well as LC3-II lipidation (Fig. 4c, d).

### Role of TAK1 in S6K1-mediated regulation of autophagy

**Fig. 5:**
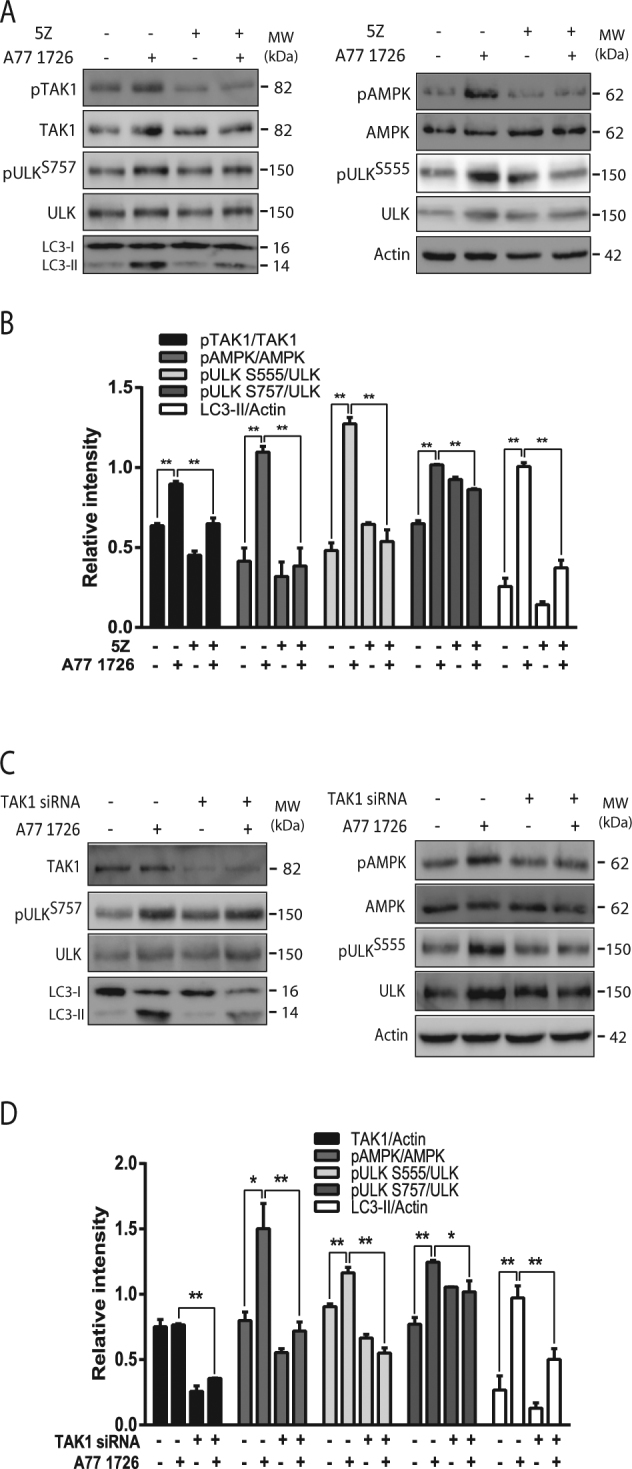
TAK1 mediates A77 1726-induced AMPK activation. NSC34 cells were treated with A77 1726 (200 μM) and/or 5Z-7-oxozeaenol (100 nM) for 16 h (**a**, **b**) or were transfected with scrambled or TAK1 siRNA (2.5 nmole each) (**c**, **d**). After incubation for 48 h, the cells were left untreated or treated with A77 1726 for 16 h. Cell lysates were prepared and analyzed for the expression of the indicated proteins by western blot. The expression levels were analyzed by quantification of the density of the protein bands with NIH Image-J software and presented as bar graphs (**c**, **d**). *p < 0.05; **p < 0.01

We next tested if S6K1 suppression by A77 1726 led to the activation of TAK1, subsequently activating AMPK. As shown in Fig. 5a, b, 5Z-7-oxozeaenol, an inhibitor of TAK1, blocked A77 1726-induced phosphorylation of TAK1^T184/187^, AMPK^T172^, and ULK1^S555^, and blocked A77 1726-induced LC3-II lipidation. TAK1 siRNA suppressed TAK1 expression (Fig. 5c, d). Consistently, suppression of TAK1 by siRNA led to the inhibition of A77 1726-induced phosphorylation of AMPK^T172^, ULK1^S555^, and TAK1^T184/187^, and blocked A77 1726-induced LC3-II lipidation (Fig. 5c, d). Both 5Z-7-oxozeaenol and S6K1 siRNA somehow also partially blocked A77 1726-induced ULK1^S757^ phosphorylation.

### Autophagy plays a critical role in A77 1726-induced SOD1 degradation

**Fig. 6:**
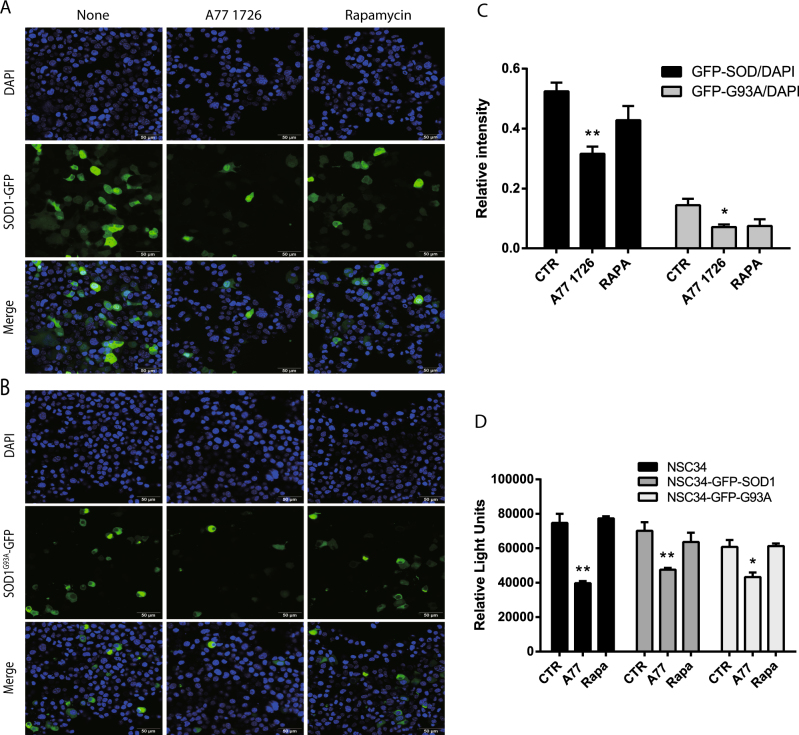
A77 1726 blocks the formation of SOD1^G93A^ protein aggregates. **a**, **b** NSC34 cells transiently transfected with SOD1-GFP or SOD1^G93A^-GFP expression vectors were treated as described in “Materials and Methods” section. The cells were examined under a confocal microscope for SOD1-GFP or SOD1^G93A^-GFP expression (**a**, **b**) and quantified for the fluorescence intensity in a plate reader (**c**). The results represent the mean ± SD from one experiment in triplicate. The experiments were repeated twice with similar results. **d** The anti-proliferative effect of A77 1726 on NSC34 cells. Untransfected NSC34 cells or the cells transfected with the SOD1-GFP or SOD1^G93A^-GFP expression vector were seeded in a 96-well plate (5000 cells per well) and incubated in the absence or presence of A77 1726 (200 μM) or rapamycin (50 nM) for 24 h. Cell proliferation was analyzed by an ATP-based Cell-Glo assay. The data are the mean ± SD of the triplicate from one representative of two experiments with similar results. *p < 0.05; **p < 0.01

A77 1726 slightly reduced the number of wild-type SOD1-GFP-positive NSC34 cells but modestly reduced the number of SOD1^G93A^-GFP-positive NSC34 cells (Fig. 6a, b). In contrast, rapamycin had little effect in the number of both wild-type SOD1-GFP and mutant SOD1^G93A^-GFP-positive NSC34 cells (Fig. 6a, b). To quantify the reduction of GFP-positive cells, NSC34 cells transfected with wild-type SOD1-GFP or mutant SOD1^G93A^-GFP expression vector in the absence or presence of A77 1726 or rapamycin were analyzed for the fluorescent intensity of the GFP-positive cells in a plate reader. As shown in Fig. 6c, the fluorescence intensity was significantly reduced in A77 1726-treated NSC34 cells transfected with either wild-type SOD1-GFP or SOD1^G93A^-GFP expression vector, compared to that in untreated controls. The fluorescence intensity was slightly reduced in rapamycin-treated NSC34 cells, but that was not statistically significant. Of note, the decreased fluorescence intensity in A77 1726-treated cells was not caused by A77 1726-mediated anti-proliferative effect since the GFP fluorescence intensity was normalized against the fluorescence intensity from the nuclear staining with 4′,6-diamidino-2-phenylindole (DAPI).

To rule out the possibility that A77 1726 reduced SOD1-GFP fluorescence intensity by selectively killing NSC34 cells expressing SOD1-GFP protein, we measured the proliferation index of untransfected NSC34 cells or NSC34 cells transfected with SOD1-GFP or SOD1^G93A^-GFP expression vector in the absence or presence of A77 1726 (200 μM) or rapamycin (50 nM). As shown in Fig. 6d, A77 1726 inhibited the proliferation of untransfected NSC34 cells slightly better than the NSC34 cells transfected with the SOD1-GFP or SOD1^G93A^-GFP expression vector. Rapamycin did not significantly inhibit NSC34 cell proliferation.

**Fig. 7:**
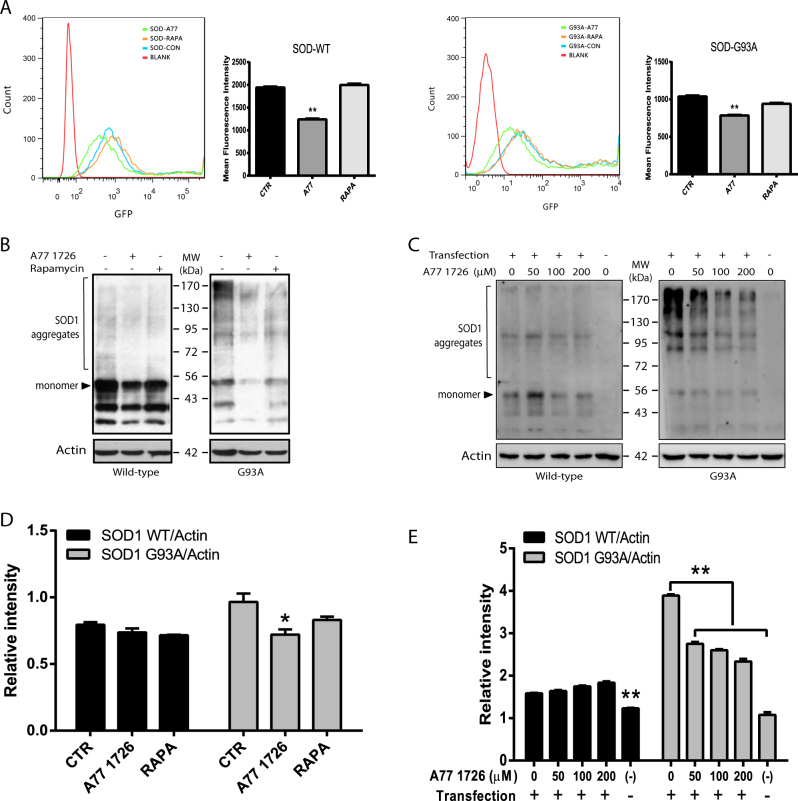
Evidence that A77 1726 induces SOD1 protein degradation. **a** NSC34 cells seeded in 60 mm dishes were transfected the SOD1-GFP or SOD1^G93A^-GFP expression vectors and treated with A77 1726 (200 μM) or rapamycin (50 nM). Single-cell suspensions were analyzed for GFP expression in a flow cytometer. The fluorescence intensity was analyzed by using FlowJo software. The results represent the mean ± SD from three independent experiments. *p < 0.05; **p < 0.01. **b**, **c** Western blot analysis of SOD1 aggregates. SOD1-GFP or SOD1^G93A^-GFP-transfected NSC34 cells were treated with A77 1726 or rapamycin as above (**b**) or treated with the indicated concentrations of A77 1726 (**c**) for 24 h. Insoluble fractions of the cell lysates were analyzed by western blot with an anti-SOD1 rabbit serum or actin. Protein aggregates as marked were analyzed by using a NIH Image J software and presented as bar graphs (**d**, **e**). *p < 0.05; **p < 0.01

Flow cytometry revealed that A77 1726 significantly shifted the peak of SOD1-GFP- and SOD1^G93A^-GFP-transfected cells to the left side; whereas rapamycin had little effect in shifting the peaks of GFP-positive cells (Fig. 7a). Western blot revealed that a very light smear of SOD1 protein aggregates was seen in wild-type SOD1-transfected NSC34 cells (Fig. 7b). In contrast, there was a very heavy smear of SOD1 mutant proteins in the insoluble fractions of GFP-SOD1^G93A^-transfected NSC34 cells (Fig. 7b). Of note, GFP-SOD1 monomer marked in Fig. 7b was detected as a heavy band with a molecular weight of ~53 kDa protein, whereas protein aggregates were detected as a dimer of ~100 kDa or multimer with heavier molecular weights. A77 1726 did not reduce wild-type SOD1 protein aggregates but reduced the smear of mutant SOD1 protein aggregates in NSC34 cells (Fig. 7b, d). Rapamycin did not significantly reduce the light smear of wild-type SOD1 aggregates nor reduced the heavy smear of mutant SOD1 aggregates in NSC34 cells (Fig. 7b, d). A77 1726 did not reduce the light smear of wild-type SOD1 protein aggregates but significantly reduced mutant SOD1 aggregates in a dose-dependent manner (Fig. 7c, e).

**Fig. 8:**
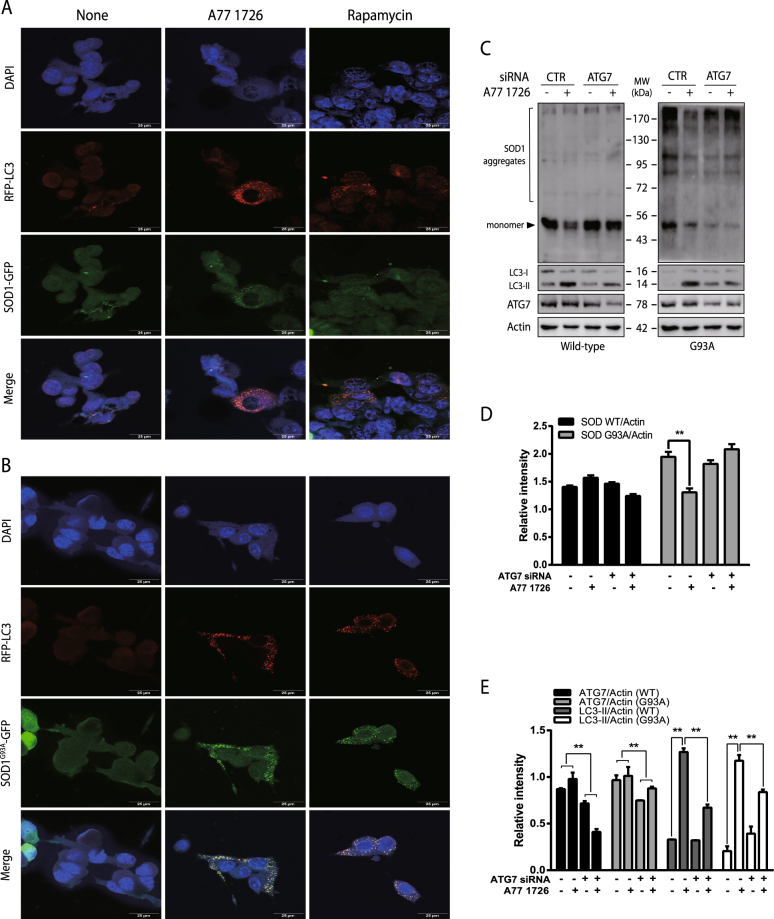
A77 1726 induces SOD1^G93A^ co-localization with autophagosomes. RFP-LC3 stably transfected NSC34 cells were transiently transfected with SOD1-GFP (**a**) or SOD1^G93A^-GFP (**b**) expression vectors. After incubation for 40 h, the cells were treated with DMSO (0.2%), A77 1726 (200 μM) or rapamycin (50 nM) (**a**) for 24 h. The cells were fixed and examined under a confocal microscope for the localization of autophagosomes (RFP-LC3) and for SOD1-GFP or SOD1^G93A^-GFP protein aggregates. **c–e** NSC34 cells were transfected with control or ATG7 siRNA (100 nmole each) and with SOD1-GFP or SOD1^G93A^-GFP expression vectors. After incubation for 48 h, the cells were collected. Cell lysates were loaded to a non-reducing gel followed by western blot analysis with indicated antibodies. The data in Fig. 8d were derived from Fig. 8c in which only the density of protein aggregates (excluding the heavy band of the 53 kDa monomer) was quantified. Data in Fig. 8e were derived from Fig. 8c in which the relative levels of ATG7 and LC3 lipidation were analyzed. *p < 0.05; **p < 0.01

Confocal microscopy revealed that both A77 1726 and rapamycin induced the formation of autophagosomes in wild-type SOD1-GFP- and mutant SOD1^G93A^-GFP-transfected NSC34 cells (Fig. 8a). RFP-LC3 autophagosomes were not co-localized with wild-type SOD1-GFP proteins in NSC34 cells in the absence or presence of A77 1726 or rapamycin. In contrast, RFP-LC3 autophagosomes were precisely co-localized with mutant SOD1^G93A^-GFP aggregates in A77 1726-treated NSC34 cells. In contrast, rapamycin induced relatively poor co-localization of RFP-LC3 autophagosomes with mutant SOD1^G93A^-GFP aggregates in NSC34 cells (Fig. 8b).

ATG7 siRNA reduced ATG7 expression by ~30% in both wild-type SOD1-GFP- and SOD1^G93A^-GFP-transfected cells (Fig. 8c, e). Suppression of ATG7 expression by ATG7 siRNA also blocked A77 1726-induced LC3 lipidation in NSC34 cells transfected with the wild-type SOD1-GFP- or SOD1^G93A^-GFP expression vector (Fig. 8c,e). ATG7 siRNA had no effect on SOD1-GFP expression levels in untreated NSC34 cells. A77 1726 had little effect on the levels of wild-type SOD1-GFP aggregates. However, A77 1726 significantly reduced the amount of SOD1^G93A^-GFP, which was blocked by ATG7 siRNA (Fig. 8c, d). Of note, the data in Fig. 8d were derived from Fig. 8c, in which only the density of protein aggregates (excluding the heavy band of the 53-kDa monomer) was quantified.

## Discussion

It is well established that mTOR phosphorylates ULK1^S757^, inhibits ULK1 activity, and suppresses autophagy. Inhibition of mTOR activity by rapamycin or nutrient starvation leads to ULK1 activation and autophagy^38, 39^. In the present study, we demonstrated that mTOR feedback activation by two S6K1 inhibitors, A77 1726 or PF-4708671, and by S6K1 siRNA did not suppress autophagy in a motoneuron cell line. Instead, S6K1 inhibitors and S6K1 siRNA induced autophagy. It appears that, even though ULK1 is phosphorylated at S757, it remains to be active in A77 1726-treated NSC34 cells, probably due to its phosphorylation at S555. Loss of function of Tuberous Sclerosis Complex 1 (TSC1) or TSC2 in the setting of the genetic condition, *Tuberous Sclerosis Complex*, activates mTORC1 and downregulates the basal level autophagy in dividing cells^40^. Interestingly, TSC2-deficient neurons with heightened mTOR activity have an efficient autophagic process through compensatory AMPK activation and increased ULK1^S555^ phosphorylation^40^.

We and others have recently demonstrated that A77 1726 and leflunomide induce autophagy in renal carcinoma and melanoma cell lines^41, 42^. Our present study focuses on the mechanisms of A77 1726-induced autophagy and its impact on degrading misfolded protein aggregates in a motor neuron cell line. A77 1726 has three biochemical activities: inhibition of pyrimidine nucleotide synthesis by inhibiting DHO-DHase activity, inhibition of PTK activities, and inhibition of S6K1 activity. Exogenous uridine was unable to block A77 1726-induced autophagy, suggesting that A77 1726-induced autophagy is independent of its inhibitory effect on pyrimidine nucleotide synthesis. Of note, the concentration of uridine used in our study was 200 μM. This uridine concentration or even lower, which has been widely reported in literature by others^43^ or by ourselves^28, 30^, is sufficient to normalize intracellular pyrimidine levels in cells treated with A77 1726 or other more potent DHO-DHase inhibitors such as brequinar sodium. Furthermore, inhibition of S6K1 activity by PF-4708671 or S6K1 siRNA included as controls also induced autophagy. A77 1726-induced autophagy is likely mediated by its inhibition of S6K1 activity. Consistent with this notion, Park et al.^44^ recently reported that PF-4708671 induces autophagy in mouse embryonic fibroblasts and promotes p62-dependent autophagic degradation of Keap1 protein.

Based on the observations that inhibition of TAK1 activity by a specific inhibitor 5Z-7-oxozeaenol and by TAK1 siRNA blocked A77 1726-induced LC3-II lipidation, we postulate that TAK1 is responsible for S6K1 inhibition-induced autophagy (Fig. 1m). In support of this notion, Kim et al.^45^ reported that S6K1 negatively regulates the activity of TAK1. Inokuchi-Shimizu et al.^27^ showed that TAK1 deficiency leads to the inhibition of starvation-induced autophagy in the liver of TAK1 knockout mice. These investigators further showed that TAK1 deficiency compromises rapamycin-induced autophagy in the hepatocytes of TAK1 knockout mice. These observations collectively suggest that TAK1 plays a key role in mediating the S6K1 inhibitor-induced autophagy (Fig. 1m).

Several studies suggest that TAK1 induces autophagy through AMPK activation. TAK1 activates AMPK-dependent cytoprotective autophagy in TRAIL-treated epithelial cells^46^. TAK1 is responsible for VEGF–induced AMPK activation in endothelial cells^47^. AMPK phosphorylation at T172 and its activity are subdued in TAK1-null embryos^26^. Consistent with these observations, our present study showed that TAK1 siRNA and 5Z-7-oxozeaenol blocked A77 1726-induced AMPK activation. The AMPK inhibitor compound C blocked A77 1726-induced ULK1^S555^ phosphorylation and autophagy. These observations collectively suggest that TAK1 plays a critical role in A77 1726-induced AMPK activation.

Previous studies have shown that S6K1 deficiency leads to AMPK activation in the skeletal muscle tissues and myotubes of S6K1-deficient mice due to increased AMP levels and AMP/ATP ratios^48, 49^. It remains to be determined if A77 1726-induced AMPK activation is in part mediated by increased AMP levels and AMP/ATP ratio. A recent study showed that Fyn tyrosine kinase phosphorylates AMPK at Y436 and suppresses its activation, as evidenced by decreased AMPK phosphorylation at T172 in TNF-α-treated HEK293 cells^50^. Our early study showed that A77 1726 is an inhibitor of the Src family tyrosine kinases p56^Lck^ and p59^Fyn^^31^. A77 1726 seemed to induce AMPK^T172^ and ULK1^S555^ phosphorylation at a lower concentration (Fig. 3a) than that required for inhibition of S6K1 activity (Fig. 1a). In addition, though PF-4708671 is more potent at inhibiting S6K1 activity than A77 1726, PF-4708671 was less effective at inducing LC3-II lipidation (Fig. 2b) and ULK1^S555^ phosphorylation than did A77 1726 in NSC34 cells. It is possible that A77 1726 may also activate AMPK and induce autophagy by inhibiting Fyn tyrosine kinase activity. Moreover, S6K1 binds to and phosphorylates AMPK α2 at S491, and inhibits AMPK activity^51^. S6K1 may regulate AMPK activity by multiple mechanisms.

In the present study, we found that A77 1726 induced autophagy and mutant SOD1^G93A^ degradation in NSC34 cells. Mutant SOD1^G93A^ aggregates were co-localized with autophagosomes. In contrast, rapamycin, though it also induced the formation of autophagosomes, had limited effect on inducing mutant SOD1 degradation as evidenced by minimal reduction of protein aggregates in western blot and fluorescent microscopic analysis. Moreover, LC3-RFP autophagosomes did not precisely co-localize with mutant SOD1 aggregates in rapamycin-treated cells. A77 1726 appears to be more effective than rapamycin in inducing SOD1 degradation. We speculate that autophagy induced by A77 1726 through AMPK activation is more robust than rapamycin-induced autophagy in motor neurons with misfolded protein aggregates. Saxena et al. reported that mTOR activation protects ALS motoneurons, delays ALS onset, and extends survival^52^. Lithium and trehalose, two AMPK activators, provide neuroprotective effects, delay ALS onset, and prolong survival in animal models^53–56^. mTOR feedback activation by S6K1 inhibitors may protect motor neurons from apoptosis. Indeed, motoneuron apoptosis is exacerbated in rapamycin-treated SOD1^G93A^-transgenic mice^57^. Recent studies showed that Src/c-Abl tyrosine kinases are highly activated in the motor neurons of ALS patients^58, 59^. Inhibition of Src expression by siRNA and activity by the Src inhibitor bosutinib induces autophagy and increases the survival of motor neurons derived from patients with SOD1^G93A^ gene mutation^58^. Our prior studies have shown that A77 1726 also inhibits the activity of the Src family tyrosine kinases p56^Lck^ and p59^Fyn^^31^. Leflunomide may function as a potent autophagy activator by targeting multiple molecules.

Leflunomide is a novel disease-modifying anti-RA drug. Its active metabolite, A77 1726, inhibits S6K1 activity with the IC_50_ values of ~50–75 μM^36^. Plasma concentrations of A77 1726 in RA patients treated with leflunomide (20 mg/day) are higher than 200 μM^60^. A77 1726 in the blood of mice treated with leflunomide at a dose of 35 mg/kg has a remarkably long half-life of 15 h. The blood concentrations of A77 1726 reached a peak of 500 μM within 4 h and remained at 250 μM at 24 h after a single dose of 35 mg/kg of leflunomide in mice^61^. Our present study showed that A77 1726 concentrations between 50 and 200 μM were very effective in inducing SOD1 mutant protein degradation (Fig. 6) and autophagy (Fig. 1). These observations suggest that the concentrations of A77 1726 used in our study are physiologically relevant. Rapamycin induced autophagy at the concentrations of nanomolar ranges, which are much lower than A77 1726 required to induce autophagy. It should be noted that the IC_50_ value required for rapamycin to inhibit its molecular target, mTOR, is also dramatically lower than the IC_50_ value of A77 1726 required to inhibit its target, S6K1. Therefore, the low IC50 values for rapamycin to induce autophagy cannot be interpreted as being more potent in inducing autophagy than A77 1726 since rapamycin and leflunomide have totally different pharmacokinetics in vivo.

In summary, our present study showed that inhibition of S6K1 activity by A77 1726 activates TAK1, leading to AMPK activation and autophagy (Fig. 1m). We further showed that A77 1726 induces SOD1 protein degradation in NSC34 cells through autophagy (Fig. 1m). Our study suggests that S6K1 can be targeted to induce autophagy, and that leflunomide may have potential to be used as a novel drug for treating ALS.

## Materials and methods

### Reagents

Leflunomide and A77 1726 were kindly provided by Cinkate Corporation (Oak Park, IL). SP600125 was purchased from Cell Signaling Technology (Danvers, MA). Rapamycin was purchased from Cayman Laboratories (Ann Arbor, MI). Bafilomycin, colchicine, metformin, 5Z-7-oxozeaenol, PF-4708671, and oligomycin were purchased from Sigma (St. Louis, MO). Anti-actin mAb was purchased from Santa Cruz Biotechnology, Inc. (Santa Cruz, CA). Antibodies against LC3, ULK1, AMPK, AKT, S6K1, S6, ACC (acetyl-CoA carboxylase) and their corresponding phospho-antibodies including ULK1^S555^, ULK1^S757^, AMPK^T172^, mTOR^S2448^, AKT^S473^, S6K1^T389^, S6^S235/236^, ACC^S79^, and TAK1^T184/187^ were purchased from Cell Signaling Technology (Danvers, MA). Anti-SOD1 antibody was kindly provided by Dr. Han-Xiang Deng (Northwestern University, Chicago). The SOD1-GFP and SOD1^G93A^-GFP expression vectors were prepared by inserting a GFP gene downstream of SOD1 in a pcDNA3.1 vector. The expression vector encoding RFP-LC3 (pmRFP-LC3) was purchased from OriGene Technologies, Inc. (Rockville, MD). The NSC34 cell line was complete DMEM medium supplemented with 10% fetal bovine serum, streptomycin and penicillin, and L-glutamine.

### Western blot

Cells grown in 6-well plates were collected and lysed in NP-40 lysis buffer (50 mM Tris-HCl (pH 8.0), 150 mM NaCl, 1% NP-40, 5 mM EDTA, 10 µg/ml aprotinin, 10 µg/ml leupeptin, and 1 mM phenylmethylsulfonyl fluoride). After incubation on ice for 30 min, the cell lysates were prepared by spinning down at 4 °C, 15,000 rpm for 15 min. For preparation of the fractions of soluble and insoluble proteins, NSC34 cells were lysed in extraction buffer (10 mM Tris-HCl pH 8.0, 1 mM EDTA, 100 mM NaCl, 0.5% NP-40, and a protease inhibitor cocktail 1:100 dilution (Thermo, Rockford, IL, USA)) followed by a brief sonication (50% output for 10 s with a probe sonicator (VCX 150, 150 W, Sonics, Newtown, CT, USA)). Cell lysates were spun down at 100,000 × *g* for 15 min at 4 °C. Pellets were resuspended in loading buffer (no β-mercapethanol) and followed by filtration through Qiagen DNA removal inserts to remove genomic DNA. Cell lysates were analyzed by western blot with antibodies against the proteins of interest, followed by horseradish peroxidase-conjugated goat anti-rabbit IgG and SuperSignal Western Pico enhanced chemiluminoscence substrate (Pierce Chemical Co., Rockford, IL). The density of the bands was analyzed by using NIH Image-J software and normalized by the arbitrary units of their corresponding total proteins or β-actin as indicated. For analysis of LC3 lipidation, the lower band of LC3-II was used to compare with β-actin. All data derived from Image-J analyses were presented as the mean ± SD from three experiments in bar graphs.

### S6K1, TAK1, and ATG7 knockdown

S6K1 siRNA ON-TARGETplus SMARTpool was synthesized by Dharmacon and purchased from Fisher Scientific (Pittsburg, PA). This S6K1 siRNA pool containing three different siRNAs has been previously shown to efficiently suppress S6K1 expression^62, 63^. TAK1 and ATG7 siRNAs were purchased from Cell Signaling Technology (Danvers, MA). A scrambled control siRNA was purchased from Life Technologies (Invitrogen Life Technologies, Grand Island, NY). NSC34 cells seeded in 6-well plates were transfected with siRNA using Lipofectamine RNAiMAX (Invitrogen Life Technologies, Grand Island, NY) according to the manufacturer’s instruction. After incubation for 48 h, the cells were collected and analyzed for the expression of S6K1 and ATG7 and other relevant proteins by western blot. To determine the effect of ATG7 on A77 1726-induced SOD1 degradation, NSC34 cells were first transfected with control or ATG7 siRNA using Lipofectamine RNAiMAX, followed by transfection with SOD1-GFP or SOD1^G93A^-GFP expression vector. After incubation for 24 h, the cells were left untreated or treated with A77 1726 for 24 h. Insoluble fractions of cell lysates were prepared and analyzed for SOD1 expression.

### Fluorescent microscopy and flow cytometric analyses of SOD1 expression

NSC34 cells were transiently transfected with an expression vector encoding the wild-type or mutant SOD1^G93A^ gene tagged with green fluorescence protein (GFP). Twenty-four hours later, SOD1-GFP and SOD1^G93A^-GFP-transfected cells were aliquoted into three wells in a 96-well plate. After incubation for 16 h, the cells were treated with dimethyl sulfoxide (DMSO) (0.2%), A77 1726 (200 μM) or rapamycin (50 nM) for 24 h. The cells were examined under a Nikon fluorescent microscope for SOD1-GFP or SOD1^G93A^-GFP expression. The cells were then fixed in methanol for 10 min at 4 °C. After air drying, the cells were replenished with 50 μl PBS per well. GFP fluorescence intensity was measured in a TECAN plate reader (Model Infinite M200 PRO) (Excitation 400 nm, Emission 508 nm). Cells were counterstained with 4′,6-diamidino-2-phenylindole (DAPI; Beyotime Institute of Biotechnology Nantong, China). The plate was then read for DAPI fluorescence intensity with excitation and emission wavelengths of 359 and 461 nm, respectively. The relative GFP fluorescence intensity = (GFP reading in each well—the mean value of GFP readings from three untransfected wells)/(DAPI reading in each well—the mean value of three blank wells). For flow cytometric analysis of GFP-positive cells, NSC34 cells were similarly transfected, aliquoted into a 6-well plate, and treated with DMSO (0.2%), A77 1726 (200 μM) or rapamycin (50 nM) for 24 h as above. Single-cell suspensions were run in a Beckman Coulter flow cytometer (Model CyAn ADP). The fluorescence intensity was analyzed by using FlowJo software. The results from three independent experiments were statistically analyzed by using the unpaired Student’s *t* test.

### Cell proliferation assay

NSC34 cells seeded in a 12-well plate (5000 cells per well) were left untransfected or transfected with the SOD1-GFP or SOD1^G93A^-GFP expression vector. After incubation for 24 h, the cells were aliquoted into a 96-well plate (5000 cells per well) and incubated overnight. The cells were then incubated in the absence or presence of A77 1726 (200 μM) or rapamycin (50 nM). After incubation for 24 h, cell proliferation was analyzed by using an ATP-based Cell-Glo assay (Promegan, Madison, WI) following the manufacturer’s instruction.

### Autophagosome analysis

NSC34 cells seeded on coverslips were transiently transfected with RFP-LC3 expression plasmid DNA using FuGENE6 following the manufacturer’s protocol. After incubation for 48 h, the cells were incubated in the presence of A77 1726 (200 μM), rapamycin (50 nM), or PF-4708671 (20 μM). After incubation for 16 h, the cells were fixed in 100% methanol at −20 °C for 10 min. The coverslips were mounted with 50% glycerin in PBS containing DAPI (0.5 μg/ml). Autophagosomes were examined under a Leica LP8 confocal microscope. The autophagosome puncta was examined under a Nikon fluorescence microscope. To determine the effect of S6K1 knockdown on autophagosome formation, NSC34 cells were transfected with control or S6K1 siRNA as described above. After incubation for 24 h, the cells were transfected with RFP-LC3 plasmid DNA again. After incubation for another 48 h, the coverslips were collected, fixed, and mounted on slides and examined for RFP fluorescence under a fluorescent microscope. Autophagosome puncta in NSC34 cells treated with various drugs or siRNA transfection were counted in 30 randomly selected fields under a 40 × objective in a blinded fashion. Results represent the mean ± SD (standard deviation) from three independent experiments. To determine whether SOD1 was co-localized with autophagosome, NSC34 cells stably transfected with RFP-LC3 was transiently transfected with SOD1-GFP or SOD1^G93A^-GFP. Twenty-four hours after transfection, the cells were treated with A77 1726 (200 μM) or rapamycin (50 nM) and then fixed and analyzed under a confocal microscope.

### Statistical analysis

An unpaired Student *t* test was used to analyze the differences in the number of puncta, the differences in the arbitrary number of western blot data from the Image J analysis, the difference in the relative light units and fluorescence intensity in NSC34 cells treated with various drugs. The data were presented as mean ± SD (western blot data, cell proliferation data, and fluorescence intensity data) or standard error of the mean (SEM) (puncta data). A *p* value of < 0.05 was considered statistically significant. All statistics was performed with SigmaPlot 11 software (Systat Software, Inc, San Jose, CA).
